# Polyurethane versus pericardium: Contrasting hemocompatibility in 2 different total artificial hearts

**DOI:** 10.1016/j.xjon.2026.101937

**Published:** 2026-06-23

**Authors:** Joseph Roux de Bezieux, Elodie Boissier, Peter Ivak, Jeanne Rancic, Aurélien Philippe, Antoine Capel, Bruno Saubamea, Christian Latrémouille, Ivan Netuka, Jean Christian Roussel, David M. Smadja

**Affiliations:** a Université Paris-Cité, INSERM U970, PARCC, Paris, France; b Carmat SAS, Velizy-Villacoublay, France; c Hematology Department, CHU de Nantes, Hôpital Nord Laënnec, Saint-Herblain, France; d Department of Cardiovascular Surgery, Institute for Clinical and Experimental Medicine, Prague, Czech Republic; e Department of Physiology, Third Faculty of Medicine, Charles University, Prague, Czech Republic; f Hematology Department, AP-HP, Georges Pompidou European Hospital, Paris, France; g Plateforme d’Imagerie Cellulaire et Moléculaire (PICMO), US25 INSERM, UAR3612 CNRS, Faculté de Pharmacie de Paris, Université Paris Cité, Paris, France; h Cardiac and Thoracic Surgery Department, CHU de Nantes, Hôpital Nord Laënnec, Saint-Herblain, France

**Figure:**
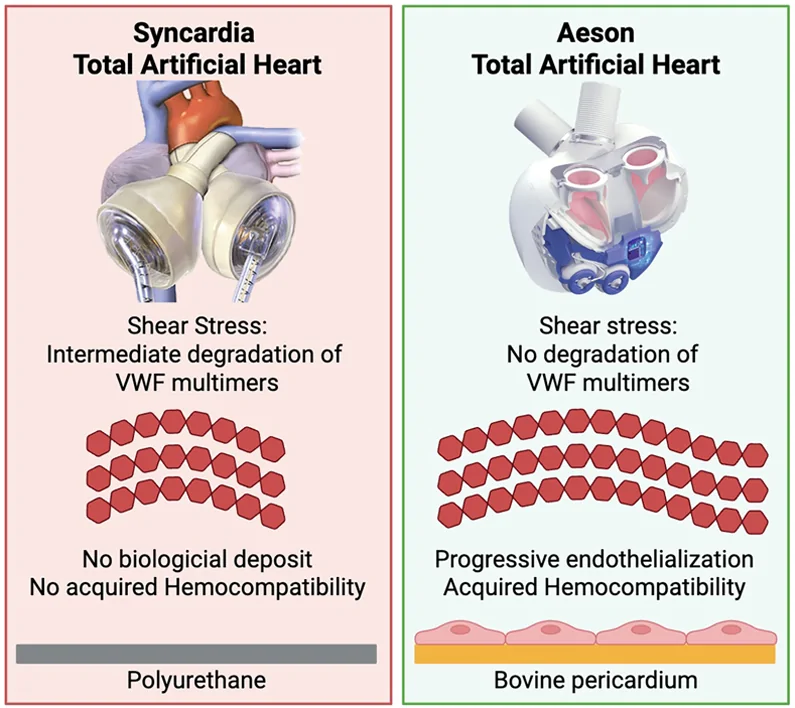
Differences between both artificial hearts in terms of hemocompatibility.

**Central Message**

Aeson total artificial heart shows greater hemocompatibility through membrane endothelialization and conservation of high molecular weight multimers of von Willebrand factor compared with SynCardia.

Mechanical circulatory support is widely used in heart failure to support or replace the failing heart. However, hemocompatibility remains a concern because of the risks of thrombosis and bleeding. Acquired von Willebrand syndrome (AVWS), linked to left ventricular assist devices, is a key contributor to bleeding, caused by shear stress–induced degradation of high molecular weight multimers (HMWMs) of von Willebrand factor (vWF), the most active form of the protein.^1^ Total artificial hearts (TAHs) replace both ventricles and generate pulsatile flow. The STAH (SynCardia) uses polyurethane (PU) for its blood-contacting surfaces. The Aeson TAH (A-TAH; CARMAT SAS), Conformité Européenne–marked in 2020, features bovine pericardium valves and membranes, with expanded polytetrafluoroethylene elsewhere. Our team previously reported no AVWS in patients with A-TAH, unlike those with HeartMate II (HM-II) or HeartMate 3 (HM-3) (both from Abbott Cardiovascular).^2^ Furthermore, the pericardium provides an acquired hemocompatibility, with preliminary results showing signs of membrane recellularization.^3^ In comparison, contradictory data exist on AVWS for patients with STAH,^4^^,^^5^ and no analysis was performed of the PU after explantation of the TAH. This study aims to assess HMWM degradation and blood-materials interaction in the STAH compared with A-TAH.

## Methods

Citrated plasma was collected from 6 patients with STAH at Nantes Hospital—3 within 1-month postimplantation and 3 after more than 3 months. Retrospective data for patients with HM-II, HM-3, and A-TAH (n = 17, clinical trial NCT02962973) were obtained from previously published studies.^2^^,^^E1^ After 3 months, the time points of patients with STAH ranged from day 149 to day 575, patients with A-TAH from day 93 to day 723, HM-II from day 97 to day 1746, and HM-3 from day 93 to day 1151. Blood samples were drawn into 0.129 mol/L trisodium citrate tubes between 2014 and 2022. vWF antigen and HMWM were measured in the same laboratory, as previously described.^2^ HMWM ratios between the A-TAH and STAH groups were compared using the Mann-Whitney *U* test, and effect size was calculated using rank-biserial correlation. Results are expressed as median [interquartile range].

One STAH and 1 A-TAH explant were analyzed in the same laboratory in 2024 and 2018, respectively, at the center of each membrane. For electron microscopy, samples were washed 3 times with 0.1 M phosphate buffer, fixed for 1 hour at 4 °C in 1% osmium and 0.1 M Sorensen's phosphate buffer (EMS) solution, then dehydrated in successive 15 minute ethanol baths of increasing concentrations, followed by Hexamethyldisilazane (MilliporeSigma). Finally, samples were glued on pads and sputtering with gold/palladium to be observed in a Jeol LV6510 scanning electron microscope. Representative images were chosen.

Institutional review board approval (2016-A00504-47-A) was obtained on June 8, 2016. Consent was obtained patients for the publication of study data.

## Results

Baseline characteristics of patients implanted with STAH and A-TAH can be found in Table 1, as well as anticoagulation and hemocompatibility-related adverse events. We evaluated the degradation HMWM of VWF in patients implanted with the STAH device at 2 distinct postimplantation periods: within the first month and after at least 3 months of support (Figure 1, *A* and *B*). The HMWM ratio in patients with STAH was consistently lower than that observed in patients with A-TAH, indicating more pronounced degradation of VWF. During the first month, the HMWM ratio was 0.81 [0.70-0.97] in patients with STAH compared with 0.99 [0.89-1.11] in patients with A-TAH (*P* = .028). After more than 3 months, the ratio remained lower in the STAH group at 0.80 [0.72-0.91], versus 0.95 [0.86-1.06] in patients with A-TAH (*P* = .0034, moderate effect size r = −0.37). Despite this significant reduction, the HMWM ratio levels in patients with STAH remained greater than those typically seen in AVWS associated with HM-II and HM-3 devices during the early postimplantation period. Interestingly, after 3 months, the HMWM ratio in patients with STAH closely approached the levels observed in patients with HM-3 devices over the same duration, suggesting a degree of biological adaptation or stabilization over time.

**Table 1:** Baseline characteristics for the patients and hemocompatibility-related adverse events (bleeding, stroke, thrombosis)

Characteristics	Patients with A-TAH	Patients with STAH
Baseline characteristics		
n	17	6
Sex, % of male	17/17 (100%)	6/6 (100%)
BMI, mean	25.8	24.8
Age, y, mean	57.9	51
Extracorporeal life support (ECMO/IABP), n	1/17 (IABP)	3/6 (ECMO)
Indication (BTT or DT), no. of BTT indications	10/17	6/6
Duration of support, median [min-max]	155 [14-772]	157 [37-859]
Anticoagulation regimen		
Anticoagulation		
Postoperative	Therapeutic unfractionated heparin: anti-Xa target 0.2-0.3 IU/mL	Therapeutic unfractionated heparin: Anti-Xa target 0.4 + coumadin (INR 2-4) for 2 patients
After renal function normalization	Tinzaparin (90-175 IU/kg/24 h) or enoxaparin (75-150 IU/kg/24 h)	
Antiplatelet agent	Aspirin	Aspirin
Adverse events		
Stroke		
Total	0/17	4/6
Ischemic	0	3
Hemorrhagic	0	1
Bleeding (excluding perioperative)		
Total patients experiencing bleeding events	29% (5/17)	83% (5/6)
Gastrointestinal event	2	4
Epistaxis event	2	0
Intracranial event	0	1
Other events	2	6
Thrombosis, type	0	1 (pulmonary embolism)

*A-TAH*, Aeson total artificial heart; *STAH*, SynCardia total artificial heart; *BMI*, body mass index; *ECMO*, extracorporeal membrane oxygenation; *IABP*, intra-aortic balloon pump; *BTT*, bridge to transplant; *DT*, destination therapy; *INR*, international normalized ratio.

**Figure 1:**
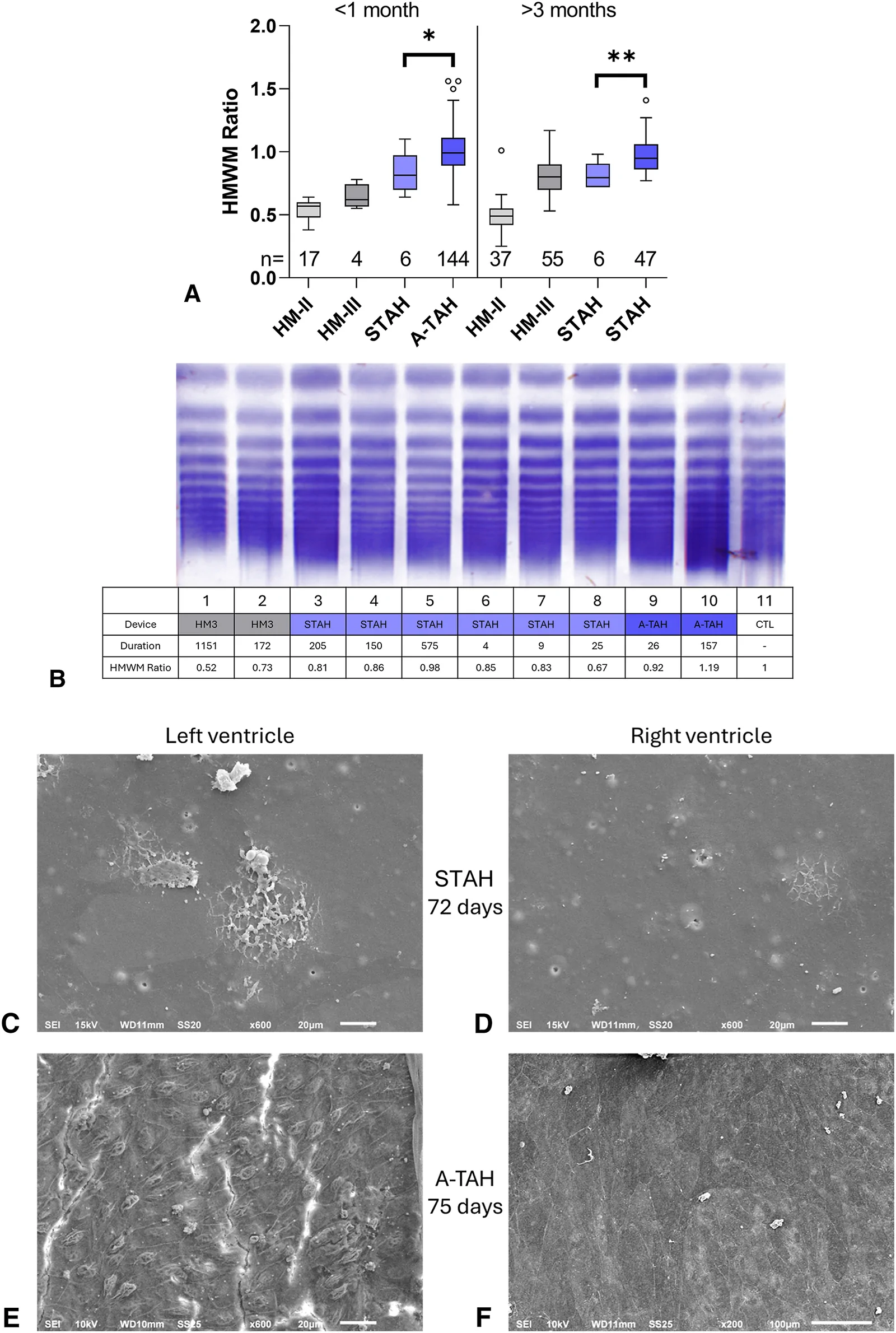
Differential von Willebrand factor (*VWF*) degradation and surface integration in polyurethane versus pericardium bioprosthetic total artificial hearts. A, High-molecular-weight multimer (*HMWM*) ratio of VWF in patients supported with HeartMate II (*HM-II*), HeartMate 3 (*HM-3*), SynCardia TAH (*STAH*), or Aeson TAH (*A-TAH*), analyzed within <1 month and >3 months after implantation. Patients implanted with STAH exhibit a significantly lower HMWM ratio compared with patients with A-TAH at both time points, indicating greater VWF degradation. Data are presented as box-and-whisker plots (median and interquartile range). ∗*P* < .05; ∗∗*P* < .01. Sample sizes are indicated below each group. B, Representative VWF multimer electrophoresis showing reduced high-molecular-weight bands in STAH samples compared with A-TAH and control (*CTL*). C and D, Scanning electron microscopy (*SEM*) of explanted STAH polyurethane membranes after 72 days of support demonstrating absence of endothelialization on left (C) and right (D) ventricular surfaces. E and F, SEM of explanted A-TAH bovine pericardial membranes after 75 days showing clear cellular coverage and endothelial-like recellularization on left (E) and right (F) ventricular surfaces. Scale bars as indicated. These findings highlight marked VWF degradation and lack of biological integration in polyurethane-based STAH compared with the endothelialized bioprosthetic A-TAH.

Explant analysis after 72 days revealed no significant biological deposition on the PU membrane of the STAH (Figure 1, *C* and *D*). This finding stands in stark contrast to the control bovine pericardium retrieved from an A-TAH device explanted after a comparable duration (75 days), which showed clear endothelialization (Figure 1, *E* and *F*). The absence of tissue coverage on the STAH membrane may reflect lower hemocompatibility of the PU material in vivo, potentially necessitating continued anticoagulation, similar to what is observed in mechanical heart valves. In contrast, the bioprosthetic properties of the A-TAH, promoting spontaneous endothelialization, could reduce the long-term anticoagulant burden, analogous to the clinical profile of bioprosthetic valves. To our knowledge, this is the first published report describing in vivo outcomes of the STAH device after patient implantation. These results offer valuable insight into the biological integration, hemocompatibility, and anticoagulation considerations associated with this next-generation TAH.

## Discussion

Two previous studies have evaluated the status of HMWM of VWF in patients implanted with the STAH. Heilman and colleagues^4^ reported no apparent decrease in HMWM in 3 patients implanted with the STAH; however, their findings were limited by the absence of quantitative analysis. In contrast, Reich and colleagues^5^ observed a moderate loss of VWF multimers in 10 of 12 patients after implantation, although this loss was not as severe as typically seen in patients supported by the HMII. Our study builds upon these findings by providing quantitative evidence that STAH implantation is associated with a significant reduction in HMWM ratio when compared with A-TAH, thereby confirming the presence of device-related VWF degradation in the STAH cohort. For SynCardia, 24.6% of patients experienced gastrointestinal (3 of 6 patients in our study) bleeding compared with 27.8% for the HM-III and 33.1% for HM-II,E2, E3 although this study focused on short implantation (median 96 days, vs 2 years for HM-III). In comparison, Only 11.8% of patients with A-TAH did have gastrointestinal bleeding during the Conformité Européenne mark study.^E1^ The comparison of HMWM ratios between devices may partly explain the observed differences in bleeding rates. All these findings should be interpreted cautiously, given differences in patient populations (noncontemporaneous cohorts from different centers) and duration of support. Furthermore, this study did not focus on other aspects such as the difference in the types of valves or the other surfaces of both TAH.

All in all, this study provides the first direct biological comparison between the SynCardia and Aeson TAHs. These preliminary results show that STAH support is associated with increased degradation of HMWM of vWF in contrast to A-TAH support, suggesting a device-related difference in shear-mediated hemocompatibility that may influence bleeding risk. Explant analyses further revealed a fundamental contrast in biomaterial behavior: bovine pericardium in the A-TAH showed endothelialization, whereas PU membranes in the STAH remained devoid of biological coverage, with potentially increased thrombotic risk and anticoagulant need. These observations need to be confirmed on more explanted devices. These observations underscore how material properties shape blood–device interactions and suggest that biomaterial-driven biological integration may be a critical determinant of hemocompatibility in next-generation artificial hearts.

## Conflict of Interest Statement

P. Ivak, I. Netuka, and D. M. Smadja received consulting fees from CARMAT SAS. J. Roux de Bezieux, C. Latrémouille, and A. Capel are employed by CARMAT SAS. All other authors reported no conflicts of interest.

The *Journal* policy requires editors and reviewers to disclose conflicts of interest and to decline handling or reviewing manuscripts for which they may have a conflict of interest. The editors and reviewers of this article have no conflicts of interest.
